# LEVERAGING COMMUNITIES AS NETWORKS TO REDUCE MALNUTRITION IN OLDER ADULTS

**DOI:** 10.1093/geroni/igz038.834

**Published:** 2019-11-08

**Authors:** Tina Sadarangani, Jeannette Beasley, Shannon E Jarrott

**Affiliations:** 1 New York University Rory Meyers College of Nursing, New York, New York, United States; 2 New York University Langone Health, New York, New York, United States; 3 The Ohio State University, Columbus, Ohio, United States

## Abstract

Malnutrition in older adults, while ubiquitous, remains largely underrecognized and undertreated. In community-dwelling older adults, 25% of those at risk of over or under nutrition do not receive any dietary interventions; routine screenings for malnutrition are not typically required in community-based settings. In this interdisciplinary symposium, we explore issues focused on the delivery of evidence-based nutrition interventions to meet the needs of community-dwelling older adults. Using national survey data, we begin by underscoring the importance of treating the complex needs of adults at risk of malnutrition by examining health sequelae, specifically hospitalizations, in community-dwelling adults receiving home-delivered meals. We subsequently examine approaches to malnutrition screening in community-based settings, focusing on the utility of the DETERMINE checklist. We explore barriers and facilitators of providing person-centered nutrition to ethnically diverse Asian American older adults in the adult day healthcare setting. Finally, we shift our focus to overnutrition, discussing the dissemination of a telehealth diabetes prevention program, BRInging the Diabetes prevention program to GEriatric populations (BRIDGE) among older adult meal program recipients. Older adults in community-based health settings are at risk of malnutrition, and among them, those who are prone to social isolation, are at highest risk for adverse outcomes. While congregate settings can facilitate social interaction, honoring food preferences and facilitating choice to address undernutrition, is challenging. Conversely, telehealth interventions may present a feasible approach for addressing overnutrition. We conclude by discussing how current and future research can inform innovative person-centered community-based approaches to identify and treat malnutrition.

